# PD229 “It’s All About Layers”: Painting The Picture Of Health Inequalities For Health Technology Assessment

**DOI:** 10.1017/S0266462324004483

**Published:** 2025-01-07

**Authors:** Robert Malcolm, Sam Woods, Hayden Holmes

## Abstract

**Introduction:**

Health inequalities can be described as avoidable, systematic, and unjust differences in health between different groups within society. This research described and evaluated potential methods to measure the effects of health inequalities that could be used in health technology assessment (HTA) in the UK. The research included recommendations for current and future policy objectives relating to incorporating health inequalities.

**Methods:**

A targeted literature review was conducted to identify methodological approaches used to incorporate health inequalities in HTA. Stakeholder interviews and a workshop were conducted with a range of stakeholders in the UK. This engagement aimed to discuss any gaps in the literature and to assess whether attitudes, methods, and policies were evolving at the same rate as the literature. Other aims of the engagement included obtaining stakeholder views on health inequalities and a better understanding of the perspectives of decision-makers.

**Results:**

Five potential methods were identified to account for health inequalities, with equity-based weighting and distributional cost-effectiveness analysis considered to be the most feasible quantification methods. Stakeholders reiterated that a deliberative process should remain the center of HTA. Stakeholders also raised issues such as the burden on committees, trade-offs between complexity and accessibility, and the importance of measuring the size and direction of inequality impacts. Recommendations were then produced based on these findings to better account for inequalities in HTA, highlighting the importance of combining a range of approaches.

**Conclusions:**

Both companies and HTA agencies should be more proactive in accounting for health inequalities. Companies should be encouraged to provide quantitative analysis on health inequalities, while decision-makers should be trained in new methods. Despite the recent rise in quantitative methods, qualitative methods remain extremely important for a layered approach to considering health inequalities.

